# Aneurismal Obstruction of Vena Cava Superior with Special Reference to the Caval Syndrome

**Published:** 1917-07

**Authors:** 


**Aneurismal Obstruction of Vena Cava Superior With Special Reference to the Caval Syndrome.** P. G. Skillern, Jr., International Clinics. The Caval Syndrome consists of enormous oedematous swelling of the head, neck, trunk, upper extremities, and marked obstruction of the veins. These clinical manifestations depend upon the formation of a collateral circulation, the extent of narrowing of the vena, and the size and extent of the pathologic process which causes the compression.

The first result of compression is obstruction of the venous blood in the entire territory of the vena cava superior. Through dilatation of all veins and capillaries in the territory of the upper half of the body an enormous cyanosis is often caused. The result of the obstructed outflow of venous blood while more blood is continually being brought to the part is the appearance of oedema. From the distribution of the oedema and its further advance one may draw diagnostic conclusions as to the site of compression. The lower half of the body is almost always free from oedema, but the latter appears here as well, when through overdistention of the inferior vena cava obstruction in the tributaries of this vein results, or when through cardiac weakness oerema appears in the lower extremities and scrotum. Usually, however, even in this case the swelling of the upper half of the body remains in characteristic contrast to the very much slighter oedema of the lower. Not only the subcutaneous cellular tissue, but also the deeper parts are involved by the oedema, especially the mediastinum. Of importance also is oedematous infiltration of the mucous membranes, for thus oedema of the glottis may give ground for suddenly appearing death.

In the **Diagnosis** of compression of the superior vena cava but little difficulty is encountered. The diagnosis is based upon the obstructive signs appearing in the territory of the vena cava superior, i.e., upon the direction of a collateral circulation and the prominence and characteristic course of the veins belonging to it. In favor of aneurism as the cause are the appearance of a dull, pulsating area and the Oliver-Cardarelli symptom.

